# Aging-associated mitochondrial circular RNAs

**DOI:** 10.18632/aging.206354

**Published:** 2026-02-10

**Authors:** Hyejin Mun, Do-Won Ham, Nam Chul Kim, Bo-In Kwon, Young-Kook Kim, Je-Hyun Yoon

**Affiliations:** 1 Department of Oncology Science, University of Oklahoma, Oklahoma City, OK 73104, USA; 2 Department of Pharmacy Practice and Pharmaceutical Science, College of Pharmacy, University of Minnesota, Duluth, MN 55812, USA; 3 Department of Pathology, College of Korean Medicine, Sangji University, Wonju-si, Gangwon-do 26339, Republic of Korea; 4 Massachusetts General Hospital, Harvard Medical School, Boston, MA 02114, USA; 5 Department of Biochemistry, Chonnam National University Medical School, Hwasun 58128, Republic of Korea; 6 Department of Pathology, University of Oklahoma, Oklahoma City, OK 73104, USA

**Keywords:** circular RNA, MT-RNR2, GRSF1, TCA cycle, aging

## Abstract

During mammalian aging, there are changes in abundance of noncoding RNAs including microRNAs, long noncoding RNAs, and circular RNAs. Although global profiles of the human transcriptome and epitranscriptome during the aging process are available, the existence and function of mitochondrial circular RNAs originating from the mitochondrial genome are poorly studied. Here, we report profiles of circular RNAs annotated to mitochondrial chromosome, chrM, in young and old cohorts. The most abundant circular RNA junctions are found in MT-RNR2, whose level is depleted in old cohorts and senescent fibroblast. The mitochondria-localized RNA-binding protein GRSF1 binds various mitochondrial transcripts, including linear and circular MT-RNR2, with a distinct RNA motif. Linear and circular MT-RNR2 bind a subset of TCA cycle enzymes, suggesting their possible function in regulating glucose metabolism in mitochondria to preserve proliferating status in young cohorts. In human fibroblasts, depletion of GRSF1 reduced levels of circMT-RNR2 and fumarate/succinate, concomitantly accelerating cellular senescence and mitochondrial dysfunction. Taken together, our findings demonstrate the existence and possible function of circular MT-RNR2 during human aging and senescence, implicating its role in promoting the TCA cycle.

## INTRODUCTION

As the ultimate goal of biomedical research, there is a great focus on increasing human lifespan and identifying the underlying molecular mechanisms behind the aging-associated decline in physiology and the elevation of pathology. Recent advances in RNA sequencing unveiled the changes of protein-coding RNAs and noncoding RNAs, along with fluctuation of RNA modifications [1–3]. The abundance of mRNAs is mainly controlled by transcription and decay via their biochemical interactions with regulatory proteins and RNAs (e.g., RNA-binding proteins and noncoding RNAs) along with cis-regulatory elements on the corresponding transcripts [3–5]. Among various regulatory noncoding RNAs, back-splicing of precursor mRNAs generates a circularized transcript, circular RNA, whose function is mainly investigated for competing with microRNAs for binding with target mRNAs [6].

Previously, we revealed RNAs that were differentially expressed during senescence of human diploid fibroblasts and aging of human Peripheral Blood Mononuclear Cells (PBMCs), impacting on differential gene expression, m6A RNA modification, and microRNA decay [3, 7]. Given the close relationship of senescence and aging in mammals, we systemically investigated m6A RNA modification regulating abundance of *AGO2* mRNA and mature microRNAs as a consequence of changes in AGO2 level [3]. Since m6A is the most abundant internal modification detected in eukaryotic mRNA, we unveiled that m6A RNA modification modulates mRNA splicing, nuclear export, localization to RNA granules, where mRNA decay and translation could be regulated [8–10]. Since the majority of m6A-modified RNAs are localized in mitochondria, transcripts transported to mitochondria from the nucleus and transcribed from the mitochondrial genome could exert critical functions in the biology of mitochondria [11].

Here, we profile circular RNA junctions from the PBMCs RNA, comparing young versus old donors to investigate the roles of mitochondrial circular RNAs, thus implicating their involvement in aging and glucose metabolism. We identified that the majority of circular RNAs are produced from a mitochondrial ribosomal RNA, MT-RNR2. Specifically, a mitochondrial RNA-binding protein, GRSF1, binds to various mitochondrial transcripts, including both linear and circular MT-RNR2. As a consequence of circular RNA production from MT-RNR2 with the help of GRSF1, the loss of MT-RNR2 also suppresses the production of mitochondrial metabolites, including fumarate and succinate, possibly by binding to a subset of TCA cycle enzymes. Global changes in the expression of mitochondrial circular RNA also suggest that MT-RNR2 depletion in old cohorts and senescent fibroblasts may be a factor regulating the TCA cycle and glucose metabolism during mammalian aging and senescence.

## RESULTS

### Profiling of circular RNA junctions from human PBMCs

**Figure 1:**
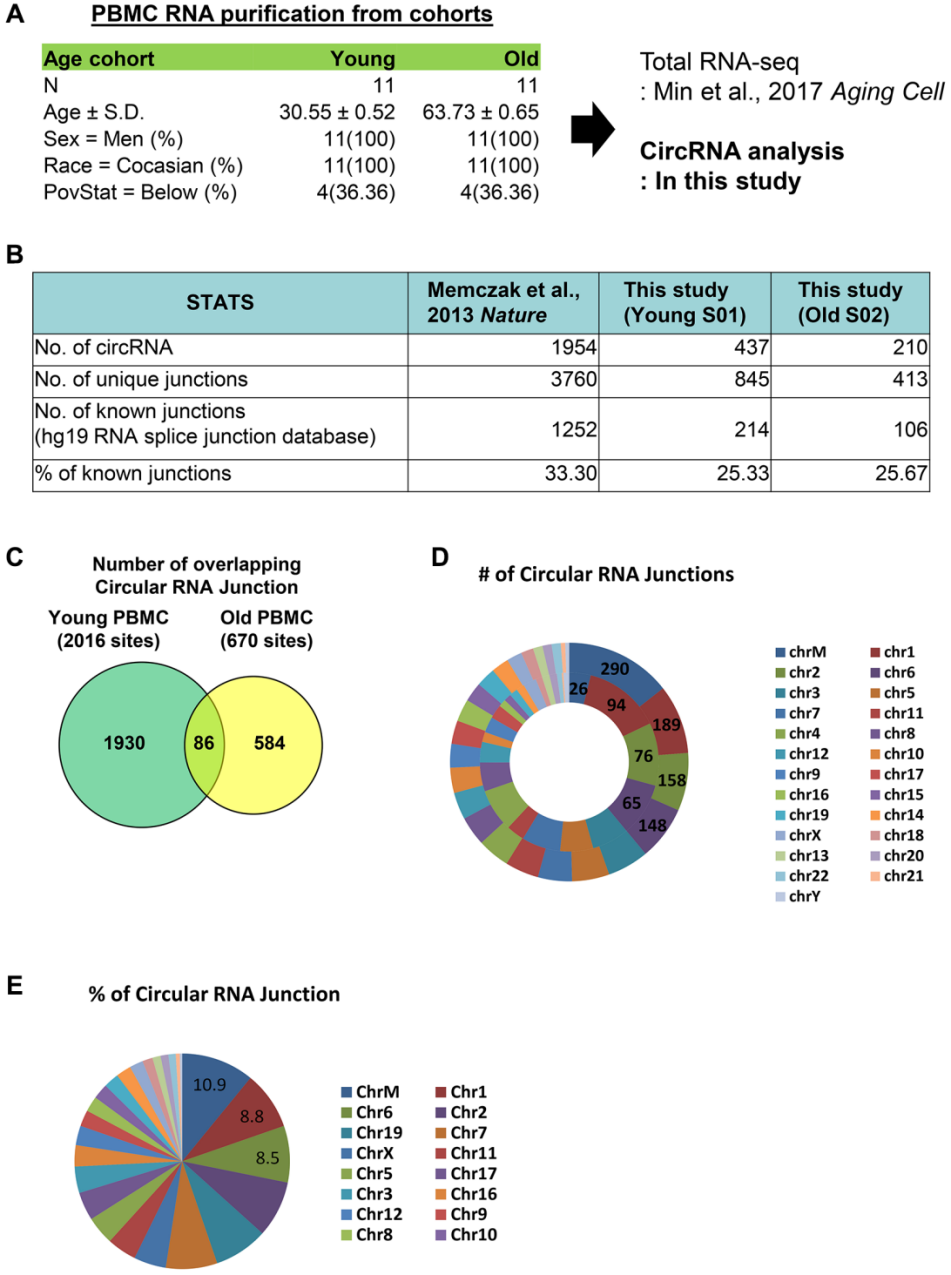
**Profiling of circular RNA junctions from human PBMCS.** (**A**) Cohorts information of PBMCs used in this study. We analyzed circular RNA junction from total RNA-seq data published previously. (**B**) Numbers of total circular RNAs, unique junctions, known junctions from previous publication and PMBCs isolated from young and old cohorts. (**C**) Common circular RNA junctions from young and old PBMCs. (**D**) Number of circular junctions originated from each chromosome. In the graph, the outer concentric circles represent data from young PBMCs and the inner concentric circles represent data from old PBMCs. (**E**) Percent of total junctions from each chromosome was calculated for the PBMCs from young cohort.

To better understand the function of circular RNA during mammalian aging, we analyzed circular RNA junctions by using total RNAs purified from 11 young and 11 old individuals (Figure 1A). Peripheral blood mononuclear cells (PBMCs) were donated by Caucasian males with the mean age of the “young” groups being 30.55 (±0.52) and, for the “old” group, 63.73 (±0.65) [3]. We purified total RNAs from the PBMCs, fragmented them, generated cDNA libraries, and sequenced them as detailed in our previous study [3].

Between the young and old groups, our analysis identified 437 and 210 circular RNAs from young and old PBMCs, respectively, which is comparable to 1954 circular RNAs from a previous publication [12]. There were 845 and 413 unique circular junctions in young and old PBMCs, and 214 and 106 junctions previously reported from hg19 RNA splice junction database. Compared to the previous publication [12], which identified 33.3% of known junctions, we have profiled 25.33% and 25.67% of known junctions from young and old PBMCs, respectively (Figure 1B). We have detected a smaller number of circular RNA species compared to previous publication, and it could be due to difference in cell types or sequencing depth.

Among total junction sites of 2016 and 670 from young and old PBMCs, 86 circular junctions overlap in both PBMCs (Figure 1C). In young PBMCs, the majority of circular RNA junctions originate from chrM, totaling 290, whereas only 26 junctions are detected from chrM in old PBMCs (Figure 1D). In young PBMCs, at least 10% of circular RNA junctions originate from chrM (Figure 1E). Taken together, our profiling of circular RNA junctions from young and old PBMCs revealed that different species of circular RNAs are differentially produced from various chromosomes, especially from chrM, in both young and old PBMCs.

### MT-RNR2 produces circular RNAs depleted in old PBMCs

**Figure 2:**
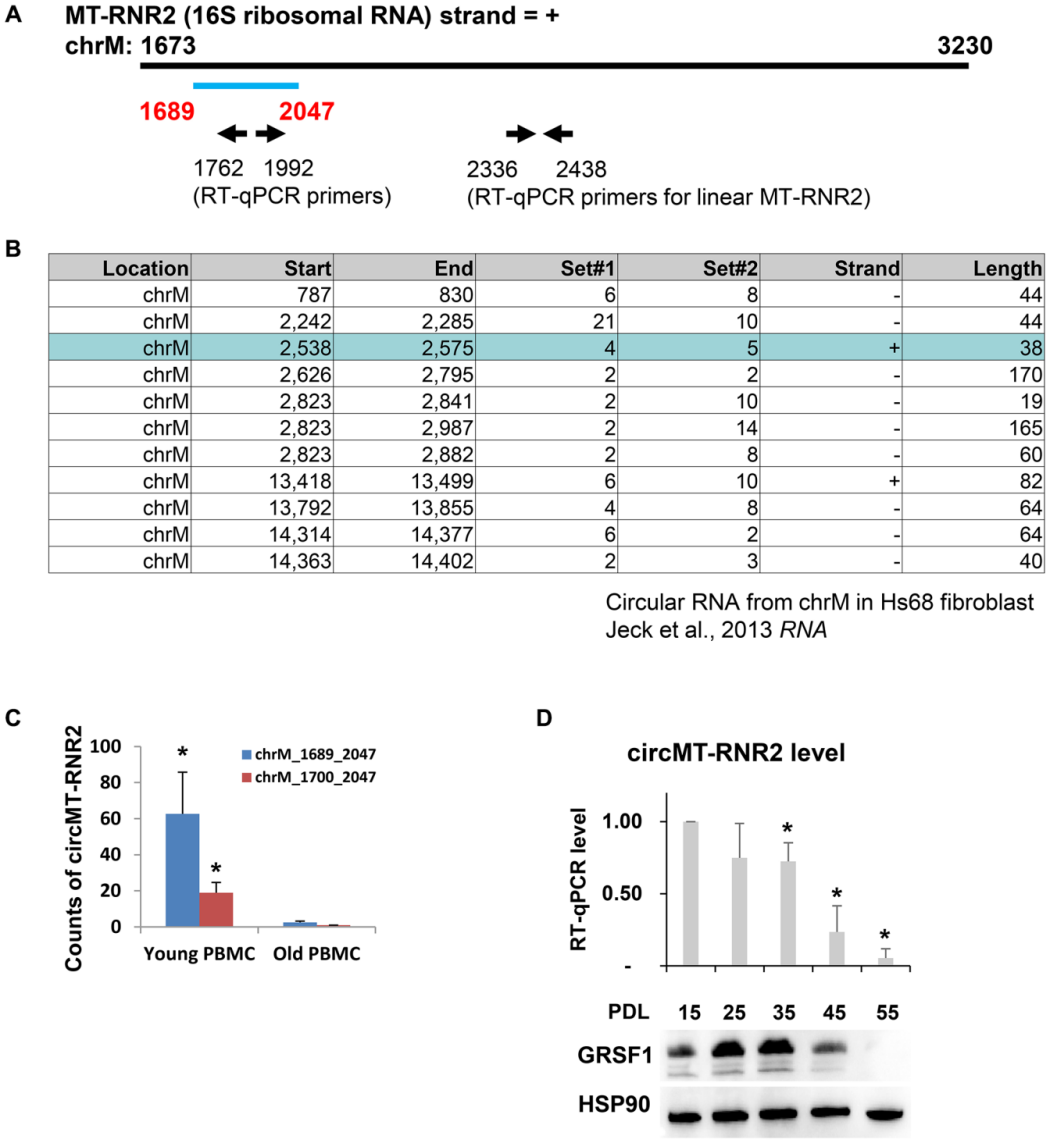
**MT-RNR2 produces circular RNAs depleted in PBMCs of old cohort and in human diploid fibroblasts, WI-38.** (**A**) Start and end of circMT-RNR2 from linear MT-RNR2. Position of primers to detect circular and linear MT-RNR2 are presented. (**B**) Circular RNA junctions identified from previous study (Jeck et al., 2013). (**C**) Abundance of circMT-RNR2 junction analyzed from total RNA-seq of PBMCs from young and old cohorts. (y-axis: count of circMT-RNR2 junction) (**D**) RT-qPCR analysis of circMT-RNR2 and western blot analysis of GRSF1 and HSP90 in WI-38 human diploid fibroblasts at different population doubling level (PDL).

From our circular RNA analysis, we have detected the most abundant circular RNAs from the mitochondrial ribosomal RNA, MT-RNR2. MT-RNR2 is produced from chrM: 1,673-3,230 from the heavy strand of human chrM. The circular RNA junction that we identified maps to 1,689-2,047 (359 nt) and 1,700-2,047 (348 nt) in respect to chrM, with no intron present within the sequence (Figure 2A). Although conventional circular RNAs are produced as a byproduct of back-splicing, this circular RNA junction might be produced by a product of trans-splicing (see the discussion). To detect circular and linear MT-RNR2, we have designed RT-qPCR primers for divergent and convergent PCR, respectively.

As previously reported in Jeck et al., there are various species of circular RNA junctions mapped to the mitochondrial genome [13]. Heavy strand of mitochondrial genome from Hs68 fibroblast produces a circular RNA junction, chrM: 2,538-2,575, within the MT-RNR2 locus with no overlap with the junction that we identified from human PBMCs (Figure 2B). In previous publication [13], the authors detected only 1 species of circMT-RNR2 (chrM:2538-2575) from positive strand while the others are produced from negative strand (including chrM:2242-2285). However, we could not detect the circular RNA from the positive strand of chrM:2538-2575 possibly due to difference in cell types or sequencing depth. When we compared the abundance of circular RNA junctions in PBMCs RNA, we observed that there is a sharp decline in the counts of 2 circular RNA junctions from PBMCs during human aging (Figure 2C). During the process of replicative senescence in human diploid fibroblasts, WI-38, the level of circMT-RNR2 declined together with senescence-suppressing RNA-binding protein GRSF1 (Figure 2D). Taken together, our analysis demonstrates that human PBMCs produce circular RNAs from MT-RNR2, which is distinct from human fibroblasts.

### Mitochondria-localized RNA-binding protein GRSF1 binds transcripts originating from the mitochondrial genome

**Figure 3:**
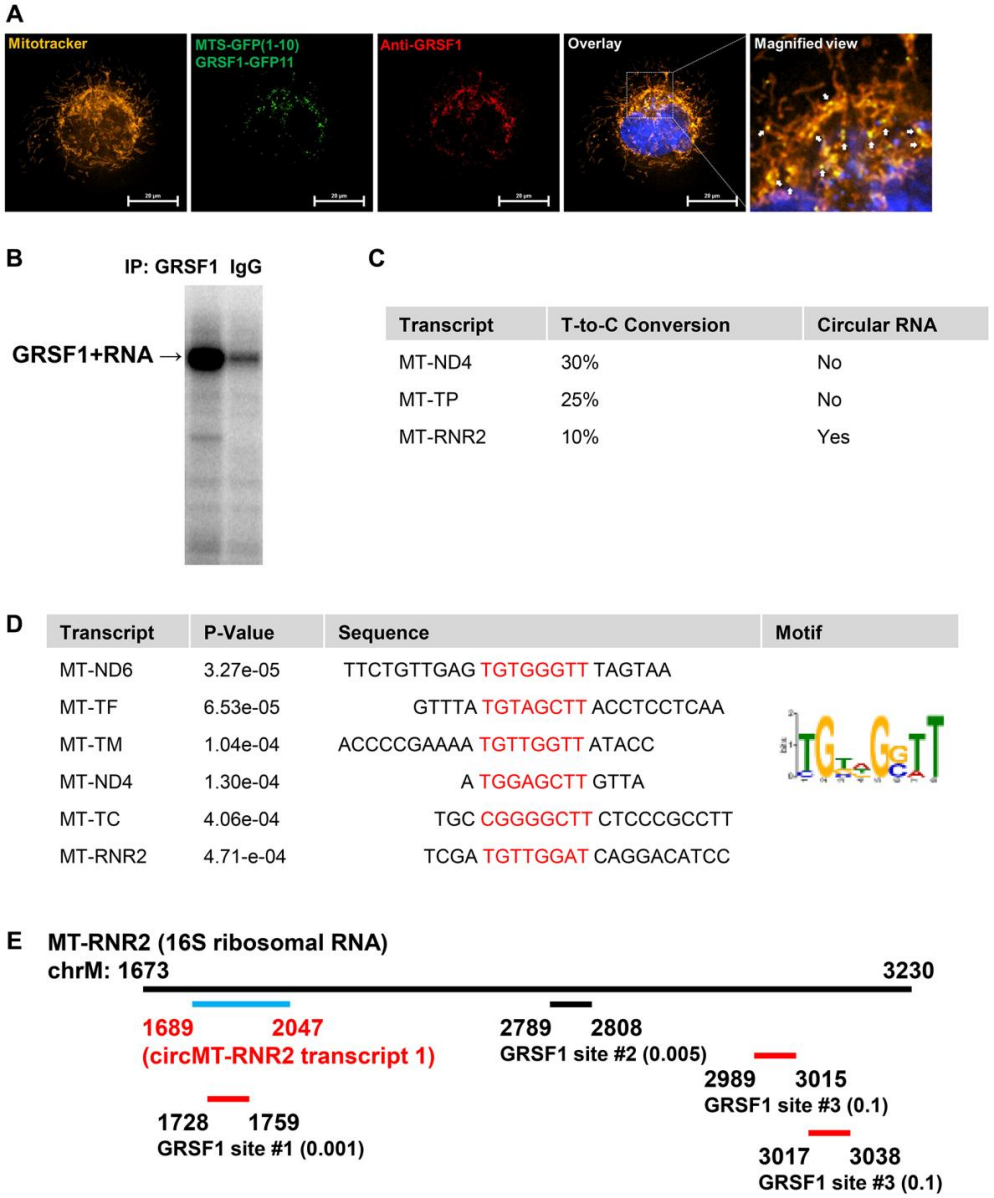
**Mitochondria-localized RNA-binding protein GRSF1 binds transcripts originated from mitochondrial genome.** (**A**) GRSF1-GFP, expressed using the split GFP system (MTS-GFP1–10/GRSF1-GFP11), localizes to mitochondria, as confirmed by co-staining with anti-GRSF1 antibody and MitoTracker. DAPI was used to stain the nucleus. White arrows in the magnified view indicate regions of GRSF1 co-localization within mitochondria. The scale bar represents 20 μm. (**B**) Autoradiograph of ^32^P-labeled RNA fragments identified from GRSF1 or IgG immunoprecipitation. (**C**) Example of GRSF1-bound mitochondrial transcripts with T-to-C conversion ratio and existence of circular RNA. (**D**) Common RNA-sequences from GRSF1 PAR-CLIP located in mitochondrial transcripts. The sequence motif and *p*-values are presented from. (**E**) Location of RNA fragments from GRSF1 PAR-CLIP located in MT-RNR2. T-to-C conversion ratio is denoted next to the coordinates.

In order to identify trans-acting factors modulating production and abundance of the circMT-RNR2, we have searched mitochondrial RNA-binding proteins interacting with MT-RNR2. Previous PAR-CLIP publications (POSTAR3) [14] reported 45 RNA-binding proteins, and there are no RNA-binding proteins localized in mitochondria. For this reason, we searched mitochondria-localized RNA-binding proteins and confirmed that a previously reported mitochondrial RNA granule marker, GRSF1 [15, 16], localized in mitochondria when we transiently expressed it using the split GFP system in HeLa cells (Figure 3A). After confirming its mitochondrial localization, we performed PAR-CLIP analysis using anti-GRSF1 antibody. Immunoprecipitation of human diploid fibroblast (WI-38) with endogenous GRSF1 using an antibody recognizing GRSF1 resulted in successful crosslinking of GRSF1 with RNA fragments, which were detected in an autoradiograph after ^32^P labeling in the SDS-PAGE. Immunoprecipitation with IgG also pulled down a certain amount of RNA fragments crosslinked with GRSF1 (Figure 3B).

The majority of PAR-CLIP tags are mapped to 3 mitochondrial transcripts, MT-ND4, MT-TP, and MT-RNR2, among which circular RNAs are produced only from MT-RNR2 (Figure 3C). Analysis of the consensus motif using the MEME suite revealed a UGxxGGUU motif present in MT-ND6, MT-TF, MT-TM, MT-ND4, MT-TC, and MT-RNR2 (Figure 3D). On MT-RNR2 locus, 4 PAR-CLIP tags are mapped; 1 inside the circular RNA region and three outside this region (Figure 3E). Thus, our findings indicate that a mitochondrial RBP, GRSF1 directly binds both linear and circular MT-RNR2, and possibly suggests that GRSF1 could be involved in the production or decay of MT-RNR2.

### MT-RNR2 binds TCA cycle enzymes

Since the abundance of mitochondrial ribosomal RNAs is closely related to mitochondrial energetics and metabolism, we have reasoned that the abundance of circular RNA produced from MT-RNR2 also could be linked to mitochondrial function. Due to biochemical interactions of circular RNAs with other noncoding RNAs, including microRNAs and RBPs for modulation of their function, we reasoned that MT-RNR2 could bind RBPs localized in mitochondria. Although we searched previous CLIP-seq data, we could not find any RBPs localized in mitochondria except GRSF1, as we investigated in this study. Because the nature of CLIP-seq restricts research to start with known RBPs, these datasets lack non-canonical RBPs that were not previously investigated in their binding to target RNAs. Those include PGK1, PGAM1, and ENO1, which we identified as binders of long noncoding RNA NEAT1 in breast cancer cells [17].

**Figure 4:**
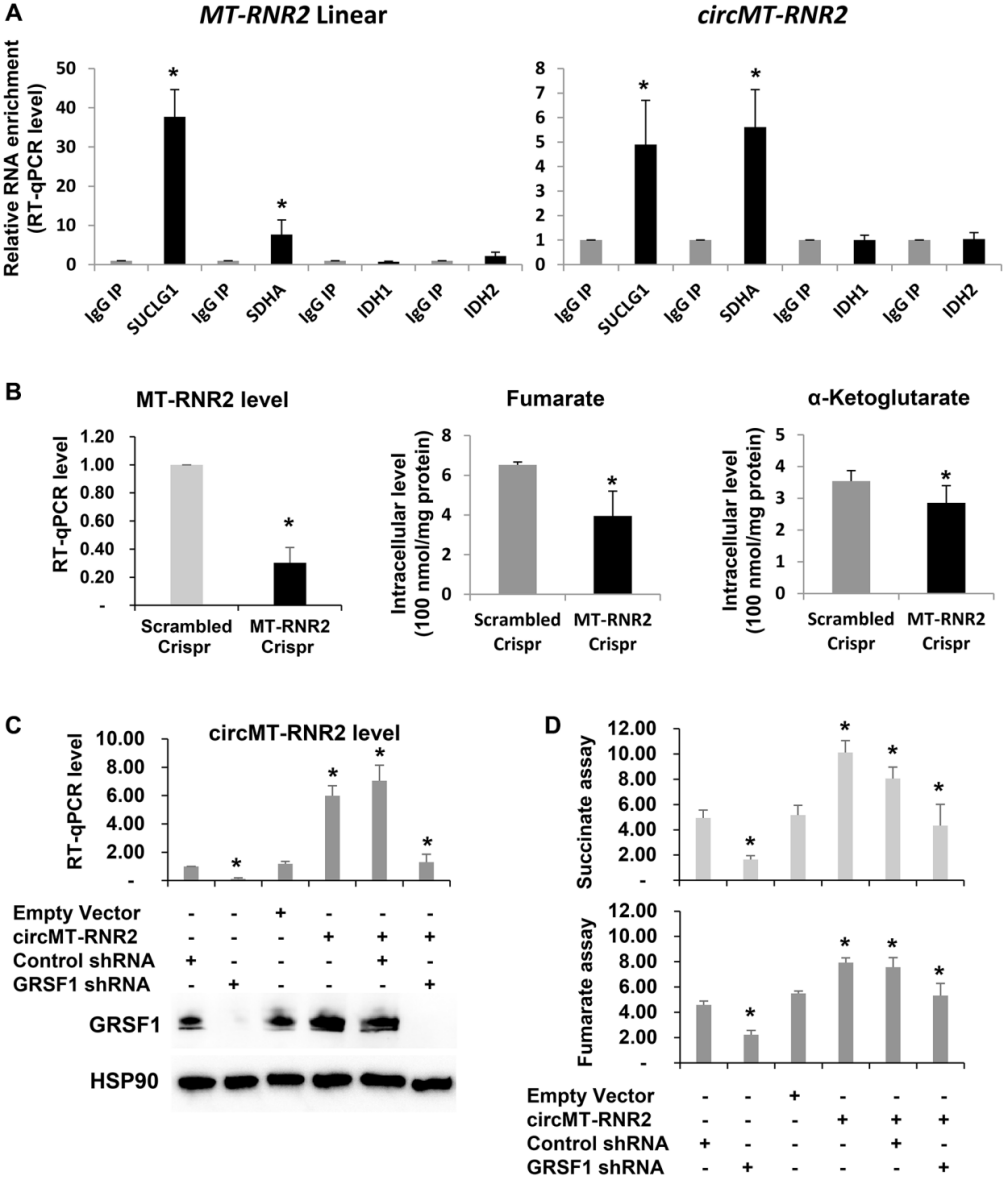
**circMT-RNR2 binds TCA cycle enzymes and accelerate production of succinate and fumarate in human diploid fibroblasts.** (**A**) RT-qPCR of RNAs purified from immunoprecipitation of IgG or antibodies recognizing SUCLG1 and SDHA. (**B**) Concentration of Fumarate and alpha-ketoglutarate after deletion of MT-RNR2 from mitochondrial genome. (**C**) RT-qPCR analysis of circMT-RNR2 and western blot analysis of GRSF1 and HSP90 in WI-38 cells after transfection with the indicated combinations of empty vector, circMT-RNR2, control shRNA, and GRSF1 shRNA. (**D**) Colorimetric levels of succinate and fumarate after transfection of circMT-RNR2 and GRSF1 shRNA plasmid.

As previously profiled from polyA-tailed RNA pull down [18–20], a series of metabolic enzymes are also pulled down together with polyA-tailed mRNAs. Those proteins include SUCLG1, SDHA, IDH1/2, and we examined their binding with linear and circular forms of MT-RNR2 from immunoprecipitation of these proteins from human fibroblast (WI-38) lysates. Our Ribonucleoprotein (RNP) immunoprecipitation assay revealed that SUCLG1 and SDHA pulled down together with both linear and circular MT-RNR2 (Figure 4A).

### circMT-RNR2 promotes TCA cycle and suppresses cellular senescence

To determine the function of circMT-RNR2 in TCA cycle and cellular senescence, we depleted MT-RNR2 in WI-38 cells and then observed decline in the concentration of fumarate and alpha-ketoglutarate as a product of the TCA cycle (Figure 4B). We also have measured the level of fumarate as a product of Succinate Dehydrogenase complex (SDHA, SDHB, SDHC, and SDHD) from succinate in the condition when circMT-RNR2 is depleted (GRSF1 shRNA) or reintroduced (circMT-RNR2 plasmid) (Figure 4C, 4D). Depletion of GRSF1 in human fibroblasts WI-38 resulted in downregulation of circMT-RNR2 and decline of fumarate level. Re-introduction of circMT-RNR2 successfully restored the level of fumarate. Level of succinate as a product of Succinyl-CoA synthetase/Succinyl-CoA ligase (SUCLG1/A1 and SUCLG2/A2 complex) also changed after GRSF1 depletion (Figure 4C, 4D). Due to limited availability of Succinyl-CoA assay system, we could not measure the level of Succinyl-CoA. These results support our conclusion that GRSF1 promotes production of circMT-RNR2 to facilitate production of fumarate and succinate as a part of TCA cycle.

**Figure 5:**
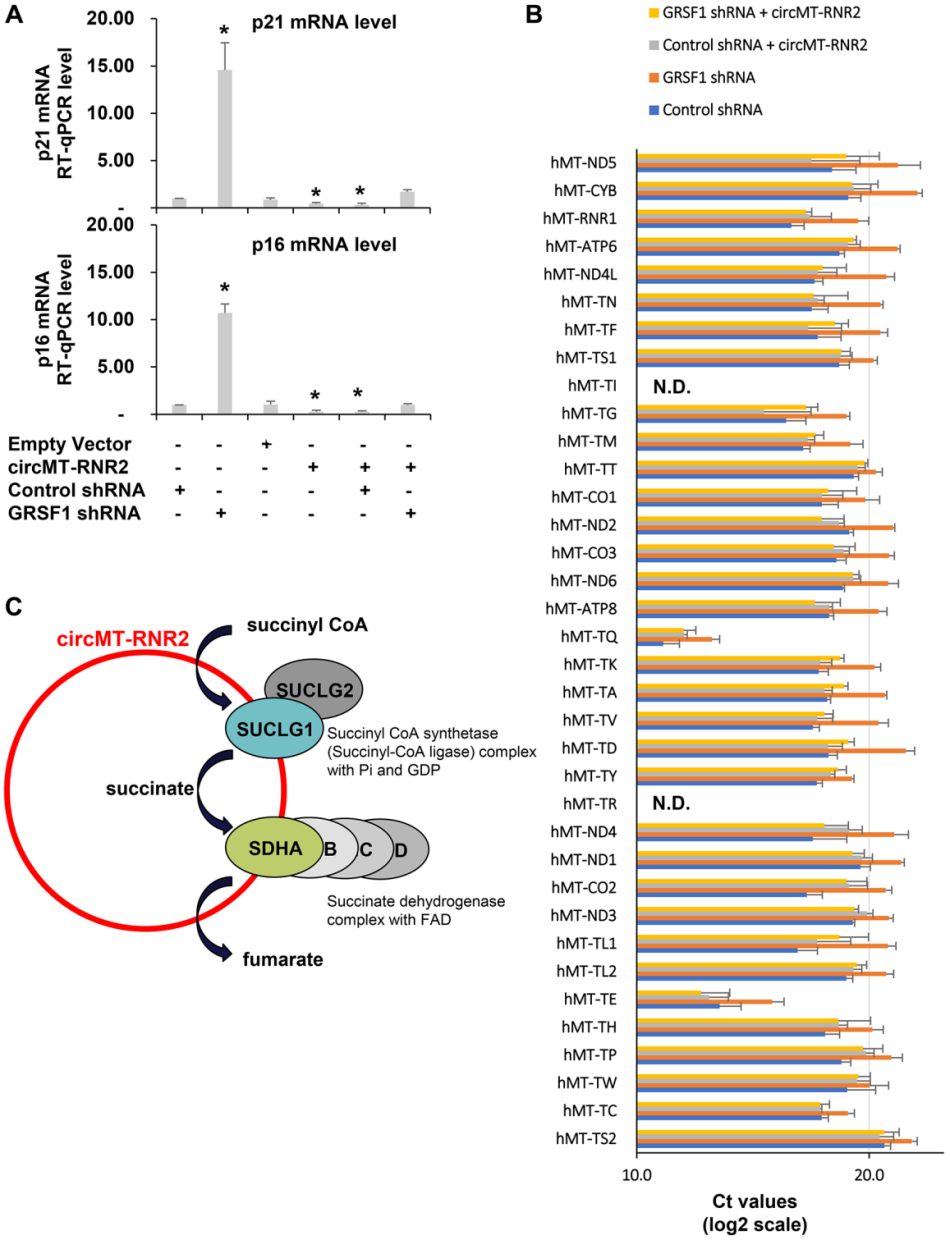
**circMT-RNR2 suppresses senescence and preserve mitochondrial homeostasis.** (**A**) RT-qPCR levels of p16 and p21 mRNAs as a marker of cellular senescence in WI-38 cells after transfection with the indicated combinations of empty vector, circMT-RNR2, control shRNA, and GRSF1 shRNA. (**B**) RT-qPCR levels of mitochondrial transcripts in WI-38 cells after transfection with the indicated combinations of empty vector, circMT-RNR2, control shRNA, and GRSF1 shRNA. Abbreviation: N.D.: Not Detected. (**C**) Possible model of circMT-RNR2 function scaffolding SUCLG1/G2 and SDHA/B/C/D to accelerate TCA cycle in mitochondria.

Since depletion of GRSF1 accelerates senescence and suppresses mitochondrial function as well as circMT-RNR2 production, we further manipulated the levels of GRSF1 and circMT-RNR2 in WI-38 cells and then compared the levels of p16/p21 mRNA as a marker of cellular senescence (Figure 5A). While GRSF1 depletion accumulates p16 and p21 mRNAs in WI-38 cells, re-introduction of circMT-RNR2 successfully rescue the level of p16 and p21 mRNAs similar to control shRNA-treated cells. In addition, GRSF1 silencing globally decreased the levels of mitochondrial transcripts but re-introduction of circMT-RNR2 partially rescued this defect in WI-38 cells as a snapshot of mitochondria homeostasis. (Figure 5B) These results demonstrate that GSRF1 facilitates production of circMT-RNR2 to accelerat TCA cycle and preserve proliferating status of fibroblasts and maintain mitochondrial function (Figure 5C). Possible molecular mechanisms will be discussed in detail below.

## DISCUSSION

Our study reveals the circular RNA profile related to human aging, the target of mitochondrial RBP GRSF1, and changes in mitochondrial metabolites following the depletion or overexpression of circMT-RNR2. Our findings above suggest that (1) depletion of circMT-RNR2 could be a driver of human aging and senescence, (2) GRSF1 is involved in circular RNA production and decay, and (3) circMT-RNR2 modulates TCA cycle by assembling metabolic enzymes.

If the expression level of circular MT-RNR2 is crucial for TCA cycle in human aging, then we should observe that there could be global changes in mitochondrial energetics in mammalian cells. We also revealed that both linear and circular MT-RNR2 co-precipitated with 2 TCA cycle enzymes. This suggests that there is a key molecular mechanism of how mitochondrial transcripts directly bind metabolic enzymes and modulate the efficiency of the TCA cycle in mitochondria. One possibility is that linear and/or circular MT-RNR2 binds multiple TCA cycle enzymes simultaneously in the same location, as long noncoding RNA NEAT1 does in breast cancer cells (Park et al., 2021). If this is a case, it will be another example of how noncoding RNAs scaffold metabolic enzymes to accelerate enzymatic reactions [21].

Since conventional circular RNAs are produced as a byproduct of back-splicing by using precursor mRNAs containing introns, it is very unique that MT-RNR2 produces circular RNAs without any introns on its loci. One scenario is that conventional spliceosome is present in mitochondria and performs its job by skipping the recognition of exon-intron junctions, which is very unlikely. Another scenario is that open-ended 5’- and 3’-ends of transcripts are ligated as a mechanism of trans-splicing, which is observed in plants and Trypanosomes [22–24]. If this is the case, a mitochondria-localized RBP, GRSF1, could be involved in this process and contribute to human aging by modulating the efficiency of the TCA cycle. Currently there is no solid report showing exact molecular mechanisms of how mitochondrial circular RNAs are generated. Although trans-splicing could be one mechanism, further studies should follow to provide more solid evidence.

GRSF1 was originally identified as a mitochondria-localized RBP, but its direct target RNAs were not identified systematically [25]. In this study, we revealed target RNAs of GRSF1 by using PAR-CLIP analysis and identified various transcripts, especially localized in mitochondria. The consensus motif recognized by GRSF1 contains a couple of Gs and Us, but does not have any repeat of Gs or Us. Deletion of GRSF1 in human fibroblasts [16–26] also revealed that GRSF1 may directly play a role in mitochondrial energetics by modulating the abundance of circMT-RNR2 during human aging and senescence. Further investigation into how GRSF1 interacts with linear and circular MT-RNR2 may provide insights into the precise mechanisms by which circular RNA regulates mitochondria.

Mitochondrial RNA granules (MRGs) are sub-mitochondrial compartments where nascent RNAs are processed, and GRSF1 has been established as a core RBP that stabilizes transcripts and coordinates their maturation with processing enzymes [15, 16]. Since GRSF1 primarily localizes to MRGs and binds to linear and circular MT-RNR2, MRGs may act as processing hubs for MT-RNR2 and other mitochondrial circular RNAs. The binding of MT-RNR2 to GRSF1 and multiple TCA enzymes further suggests that MRGs may play a role in metabolic processes, or that the scaffolding of multiple RNA-binding proteins by these RNAs underlies how MRGs form in mitochondria, potentially via liquid-liquid phase separation.

Taken together, our observations demonstrated the existence and function of mitochondrial circular RNA in mitochondrial metabolism and human aging as well as senescence. Future mechanistic studies will reveal how these mitochondrial circular RNAs are produced by trans-splicing, possibly, and how the circular RNAs accelerate the TCA cycle to preserve the proliferation status and suppress senescence as well as aging.

## MATERIALS AND METHODS

### Human study participants

For RNA profiling, a subcohort of young (~30 years) and old (~64 years) participants from the “Healthy Aging in Neighborhoods of Diversity across the Life Span study” (HANDLS) [3, 27–29] was chosen for examination of total transcriptome and circular RNAs. Clinical information on this subcohort has been described previously and is also listed in Figure 1 (*n* = 11/group) [28, 29]. The HANDLS study is approved by the Institutional Review Board of the National Institute of Environmental Health Sciences, National Institutes of Health. All of the participants signed a written informed consent document.

### Cell culture, transfection, and plasmids

Human WI-38 cells (PDL-15) were cultured in DMEM (Invitrogen) supplemented with 10% (v/v) FBS and antibiotics, and were continuously passed to reach PDL-55. Plasmids were transfected at 1–2 μg/ml [Control sgRNA, MT-RNR2 sgRNA, Control shRNA, GRSF1 shRNA, pcDNA3 empty vector, or circMT-RNR2 in the backbone of pcDNA3.1(+) HIPK3 from J.E. Wilusz Lab, Addgene#60634]. Transfected cells were analyzed 48 h later for total RNA isolation, western blot analysis, and metabolite measurement. Transcript levels were normalized to the abundance of GAPDH mRNA.

HeLa cells were obtained from ATCC and maintained in DMEM (GenClone) supplemented with 10% FBS (Gibco), 1% penicillin-streptomycin (Gibco), and 1% GlutaMAX (Gibco). Cells were cultured at 37°C in a humidified incubator with 5% CO_2_ and seeded on 35-mm glass-bottom dishes (Cellvis) one day prior to transfection. Transient transfection was performed using JetPRIME reagent (Polyplus) according to the manufacturer’s instructions. For split-GFP experiments, cells were co-transfected with MTS-GFP1-10 modified from MTS-mCherry-GFP1-10 (Addgene#91957), which localizes to mitochondria, and pcDNA3.1-GRSF1-GFP11 to confirm the mitochondrial localization of GRSF1 for 24 h at 37°C.

### Immunofluorescence assay

HeLa cells transfected with MTS-GFP1–10 and GRSF1-GFP11 were stained with 100 nM MitoTracker^™^ Deep Red FM (Invitrogen) in culture medium at 37°C for 30 min to visualize mitochondria. After rinsing with PBS, cells were fixed with 4% paraformaldehyde in PBS for 15 min at room temperature (RT), permeabilized with 0.3% Triton X-100 in PBS for 10 min and blocked with 3% bovine serum albumin (BSA) in PBS for 30 min. Cells were incubated for 2 hours at RT with a rabbit polyclonal anti-GRSF1 antibody (Atlas Antibodies; 1:200) diluted in 3% BSA in PBS. After rinsing with PBS three times, Alexa Fluor 568–conjugated anti-rabbit secondary antibody (Invitrogen; 1:200) was applied for 1.5 hours at RT. After rinsing with PBS three times, nuclei were counter-stained with DAPI (4′,6-diamidino-2-phenylindole; 2 μg/mL) for 5 min at RT. Samples were mounted with 30 μL of Fluoromount-G (SouthernBiotech) prior to imaging. Confocal images were acquired using a Nikon Eclipse Ti2 inverted microscope equipped with a Crest Optics X-Light V2 (L-FOV) spinning disk confocal unit and a 100× silicone oil immersion objective. Fluorescent signals from DAPI, GFP (split-GFP complementation), Alexa Fluor 568, and MitoTracker Deep Red were captured using appropriate filter sets. Images were acquired, processed, and overlayed using NIS-Elements software (Nikon Instruments) for figure presentation.

### RT-qPCR analysis

Total RNA was isolated, and cDNA was generated from 0.5 μgs of RNA using random hexamers and reverse transcriptase (Maxima, Thermo Scientific). qPCR was carried out using SYBR green master mix (Kapa Biosystems), and a thermal cycler (Bio-Rad). The primers used for RT-qPCR are provided in Table 1. The relative quantities of mRNAs were calculated using the ΔΔCt method and normalized using human GAPDH mRNA as endogenous control.

### Circular RNA analysis

Circular RNA junctions were analyzed as we performed previously. After removing low-quality reads from raw FASTQ sequencing files using Trimmomatic [30], chimerically aligned reads from STAR aligner [31] were used to calculate the count of circular RNA junction spanning reads based on DCC algorithm [32]. The counts for each circular RNA junction were normalized based on the total circular RNA counts in each sample.

### Western blot analysis

Whole-cell lysates, prepared in radioimmunoprecipitation assay (RIPA) buffer, were separated by sodium dodecyl sulfate-polyacrylamide gel electrophoresis (SDS-PAGE) and transferred onto nitrocellulose membranes (Invitrogen iBlot Stack). Primary antibodies recognizing SUCLG1, SDHA, IDH1, IDH2, and Actin were purchased from Cell Signaling Technology. Anti-GSRF1 antibody is from Sigma and anti-HSP90 antibody is from Santacruz Biotechnology. Horse Radish Peroxidase (HRP)-conjugated secondary antibodies were purchased from GE Healthcare.

### PAR-CLIP analysis

PAR-CLIP analysis was performed as we published previously [33, 34]. 1 × 10^8^ RAW HEK cells were incubated in medium supplemented with 100 μM 4-thiouridine (4SU) for 16 h. The cells were washed with PBS, and irradiated with 0.15 mJ cm^−2^ and 365 nm ultraviolet (UV) light in a UV crosslinker (Spectrolinker XL-1500) to crosslink the labeled RNA to RNA-binding proteins. The lysates were treated with 1 U ml^−1^ RNase T1 (Fermentas) and GRSF1 proteins were immunoprecipitated with anti-GRSF1 antibodies and Protein A/G-sepharose beads. The immunoprecipitates were further digested with 100 U ml^−1^ RNase T1 and the beads were washed in lysis buffer and suspended in one bead volume of dephosphorylation buffer. RNAs were dephosphorylated and radioactively labeled with [γ-^32^P]-ATP. The protein-RNA complexes were separated by SDS-PAGE and visualized by autoradiography. The radioactive bands were eluted from the gel and all proteins in eluted bands were removed by digestion with 0.2 mg ml^−1^ proteinase K. The RNAs were isolated by acidic phenol/chloroform extraction and ethanol precipitation, converted into a cDNA library, and sequenced using an Illumina platform (HiSeq2500). Processed reads were aligned to the reference genome (hg19) by the Bowtie algorithm, allowing for two alignment errors (mutation, insertion, or deletion). For each read, only the best mapping was reported out of a maximum of 10 genomic matches. After the conversion subtraction, reads that mapped to only one genomic location were retained for further analysis.

### RNA immunoprecipitation

RNA immunoprecipitation (RIP) analysis from whole cell extracts was performed as described previously [34–36]. Briefly, cells were lysed in buffer containing 20 mM Tris-HCl (pH7.5), 100 mM KCl, 5 mM MgCl_2_, and 2.0% NP-40, then cleared by centrifugation. The lysates were incubated with protein A-Sepharose beads coated with antibodies against GRSF1 (Millipore) with control IgG (SCBT) at 4°C for 1 h. After the beads were washed four times with NT2 buffer [50 mM Tris-HCl (pH 7.5), 150 mM NaCl, 1 mM MgCl_2_, 0.05% NP-40], the immunoprecipitates were treated with 20 units of RNase-free DNase I at 37°C for 15 min and proteinase K (0.5 mg/ml) in 0.1% SDS at 55°C for 15 min to remove DNAs and proteins, respectively. The RNAs isolated from the IPs by acidic phenol extraction were then subjected to RT-qPCR using the primers listed in Table 1. The RIP results were normalized to *GADPH* mRNA.

**Table 1:** Primer sequences.

**Name**	**Species**	**Sequence**
GAPDH F	Human	AGCCACATCGCTCAGACAC
GAPDH R	Human	GCCCAATACGACCAAATCC
p16 F	Human	GTA AGC CAA CCG ATG GAG AA
p16 R	Human	ACA CAT GTG GGT TTG CTG AA
p21 F	Human	GAC ACC ACT GGA GGG TGA CT
p21 R	Human	CAG GTC CAC ATG GTC TTC CT
MT-RNR2 F	Human	C A T A A G C C T G C G T C A G A T C A
MT-RNR2 R	Human	C C T G T G T T G G G T T G A C A G T G
MT-RNR2-C F	Human	T G G T G A T A G C T G G T T G T C C A
MT-RNR2-C R	Human	C T A T T G C G C C A G G T T T C A A T
MT-RNR2-F F	Human	A G C C A A A G C T A A G A C C C C C
MT-RNR2-F R	Human	C T C C T T G C A A A G T T A T T T C T A G T T A A T
hMT-TS2 F	Human	AAGCTCACAAGAACTGCTAAC
hMT-TS2 R	Human	AGAAAGCCATGTTGTTAGACATG
hMT-TR F	Human	GTATATAGTTTAAACAAAA
hMT-TR R	Human	GTAAATATGATTATCATAA
hMT-TT F	Human	TTGTAGTATAAACTAATACACCAGT
hMT-TT R	Human	TCCTTGGAAAAAGGTTTTCAT
hMT-TC F	Human	CTCCGAGGTGATTTTCATATTGAA
hMT-TC R	Human	CCCGGCAGGTTTGAAGCT
hMT-TY F	Human	TTAGGTTAAATACAGACCAAGAGC
hMT-TY R	Human	GAAATTAAGTATTGCAACTTACTGAGG
hMT-TM F	Human	GGTCAGCTAAATAAGCTATCGG
hMT-TM R	Human	GTACGGGAAGGGTATAACCA
hMT-TW F	Human	TTAGGTTAAATACAGACCAAGAGC
hMT-TW R	Human	GAAATTAAGTATTGCAACTTACTGAG
hMT-TD F	Human	AGAAAAACCATTTCATAACTTTGTCA
hMT-TD R	Human	AAGATATATAGGATTTAGCCTATAATT
hMT-TG F	Human	AGTATAAATAGTACCGTTAACTTCCA
hMT-TG R	Human	ACTCTTTTTTGAATGTTGTCAAAA
hMT-TP F	Human	ATAGTTTAAATTAGAATCTTAGCTTT
hMT-TP R	Human	AGAGAAAAAGTCTTTAACTCCAC
hMT-TV F	Human	GAGTGTAGCTTAACACAAAGCA
hMT-TV R	Human	GGTCAAGTTAAGTTGAAATCTCCT
hMT-TI F	Human	AATATGTCTGATAAAAGAGTTACTT
hMT-TI R	Human	TAAGGGGGTTTAAGCTCC
hMT-TH F	Human	AATATAGTTTAACCAAAACATCAGAT
hMT-TH R	Human	TAAGGGGTCGTAAGCCTC
hMT-TA F	Human	GGGCTTAGCTTAATTAAAGTGGC
hMT-TA R	Human	TGCAAAACCCCACTCTGC
hMT-TS1 F	Human	AAGTCATGGAGGCCATGG
hMT-TS1 R	Human	AAGGAAGGAATCGAACCCC
hMT-TE F	Human	TTGTAGTTGAAATACAACGATGGT
hMT-TE R	Human	TCGCACGGACTACAACCA
hMT-TK F	Human	AGCTAACTTAGCATTAACCTTTTAAGT
hMT-TK R	Human	ACTGTAAAGAGGTGTTGGTTCT
hMT-TF F	Human	TATGTAGCTTACCTCCTCAAAGC
hMT-TF R	Human	GGGTGATGTGAGCCCGTC
hMT-TL2 F	Human	TTTAAAGGATAACAGCTATCCATTGG
hMT-TL2 R	Human	TTTGGAGTTGCACCAAAATTTTT
hMT-TQ F	Human	GATGGGGTGTGATAGGTGG
hMT-TQ R	Human	TGAGAATCGAACCCATCCC
hMT-TN F	Human	AGATTGAAGCCAGTTGATTAGG
hMT-TN R	Human	GGACTTAAACCCACAAACAC
hMT-TL1 F	Human	TAAGATGGCAGAGCCCGG
hMT-TL1 R	Human	TTGAACCTCTGACTGTAAAGTTTT
hMT-ATP8 F	Human	TAAATACTACCGTATGGCCCAC
hMT-ATP8 R	Human	GTGATGAGGAATAGTGTAAGGAG
hMT-ND4L F	Human	CTCATAACCCTCAACACCCA
hMT-ND4L R	Human	AGACTAGTATGGCAATAGGCAC
hMT-ND3 F	Human	TTGATCTAGAAATTGCCCTCC
hMT-ND3 R	Human	GGCAGGTTAGTTGTTTGTAGG
hMT-ND6 F	Human	GCTTTGTATGATTATGGGCGT
hMT-ND6 R	Human	GGCAGGTTAGTTGTTTGTAGG
hMT-ATP6 F	Human	TCCCTCTACACTTATCATCTTCAC
hMT-ATP6 R	Human	GACAGCGATTTCTAGGATAGTC
hMT-CO2 F	Human	ACGCATCCTTTACATAACAGAC
hMT-CO2 R	Human	GCCAATTGATTTGATGGTAAGG
hMT-CO3 F	Human	CTCTCAGCCCTCCTAATGAC
hMT-CO3 R	Human	GCGTTATGGAGTGGAAGTG
hMT-RNR1 F	Human	AAGATTACACATGCAAGCATCC
hMT-RNR1 R	Human	TTGATCGTGGTGATTTAGAGG
hMT-ND1 F	Human	TCCTACTCCTCATTGTACCCA
hMT-ND1 R	Human	TTTCGTTCGGTAAGCATTAGG
hMT-ND2 F	Human	GTAAGCCTTCTCCTCACTCTC
hMT-ND2 R	Human	TTTCGTTCGGTAAGCATTAGG
hMT-CYB F	Human	ATCACTTTATTGACTCCTAGCC
hMT-CYB R	Human	TGGTTGTCCTCCGATTCAG
hMT-ND4 F	Human	CCCTTCCTTGTACTATCCCT
hMT-ND4 R	Human	TTTGTCGTAGGCAGATGGAG
hMT-CO1 F	Human	ATATTTCACCTCCGCTACCA
hMT-CO1 R	Human	TTTGTCGTAGGCAGATGGAG
hMT-RNR2 F	Human	AACTCGGCAAATCTTACCC
hMT-RNR2 R	Human	AATACTGGTGATGCTAGAGGTG
hMT-ND5 F	Human	TCTTAGTTACCGCTAACAACC
hMT-ND5 R	Human	ATAATTCCTACGCCCTCTCAG

### Colorimetric measurement of mitochondrial metabolite

Cellular levels of fumarate, succinate, and alpha-ketoglutarate were measured by using a commercial kit provided by Sigma and Abcam respectively. For the assay, we lysed the cells by using a buffer containing 20 mM Tris-HCl (pH7.5), 100 mM KCl, 5 mM MgCl2, and 2.0% NP-40, then cleared by centrifugation. The lysates were incubated with reaction mixtures for fumarate, succinate, or alpha-ketoglutarate, to generate a product quantified by absorbance at 570 nm.

### Quantification and statistical analysis

Data are expressed as the mean +/− S.D. of the values from at least three independent experiments performed, as indicated in the corresponding figure legends. The numbers of biological replicates, and what they represent, are indicated in each figure legend. Two-tailed Student’s *t*-tests were used for single comparison, and *p*-values below 0.05 were considered statistically significant.

### Data availability/availability of data and materials

Data and materials supporting the findings of this study are available upon request.
